# Lymphangiome kystique de la parotide

**DOI:** 10.11604/pamj.2015.20.443.6300

**Published:** 2015-04-30

**Authors:** Khaled Khamassi, Madiha Mahfoudhi

**Affiliations:** 1 Service ORL, Hôpital Charles Nicolle, Tunis, Tunisie; 2 Service de Médecine Interne A, Hôpital Charles Nicolle, Tunis, Tunisie

**Keywords:** Lymphagiome kystique, parotide, chirurgie, cystic lymphangioma, parotide, surgery

## Image en medicine

Le lymphangiome kystique est une tumeur bénigne en rapport avec une dysembryoplasie portant sur le système lymphatique. Rare chez l'adulte, il survient généralement avant l’âge de deux ans. Son siège primitif au niveau de la parotide est rarement rapporté dans la littérature. Bien que bénigne, il peut être potentiellement grave par sa tendance extensive et infiltrante des tissus de voisinage et par ses complications aigues telles que les surinfections, les poussées inflammatoires et hémorragiques. La chirurgie conservatrice est l'approche la plus souvent recommandée. Patient âgé de 15 ans sans antécédents particuliers a consulté pour une masse parotidienne droite évoluant depuis deux mois. L'examen physique a retrouvé une tuméfaction parotidienne droite de 40 mm de grand axe, bien limitée, homogène, dépressible, molle, mobile, indolore et recouverte d'une peau saine. A la biologie, il n'avait pas de syndrome inflammatoire. L’échographie cervicale a objectivé une masse ovalaire de 43 x 20 mm avec un aspect hypoéchogène, hétérogène, contenant des plages liquidiennes. La TDM cervicale a révélé une formation parotidienne inférieure droite d'allure kystique. Les diagnostics d'un lymphome, un lymphangiome ou un cancer de la parotide ont été évoqués. L'IRM cervicale a conclut à une formation kystique multiloculée contenant des niveaux liquides se développant au dépens de la parotide correspondant à un lymphangiome kystique du pôle inférieur de la parotide compliquée d'une hémorragie intrakystique. Le traitement s'est basé sur une parotidectomie inférieure emportant le lymphangiome. Les suites opératoires étaient simples. L'examen anatomopathologique a confirmé le diagnostic du lymphangiome kystique.

**Figure 1:**
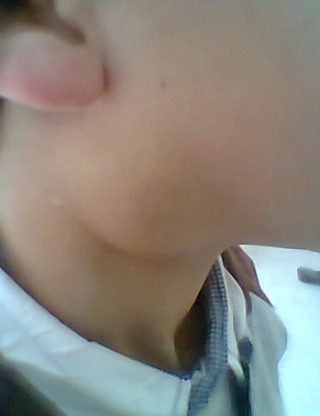
Tuméfaction ovalaire de 40 mm de grand axe en regard du pôle inférieur de la parotide droite

